# Assessment of Agreement Between a New Application to Compute the Wisconsin Gait Score and 3-Dimensional Gait Analysis, and Reliability of the Application in Stroke Patients

**DOI:** 10.3389/fnhum.2022.775261

**Published:** 2022-02-03

**Authors:** Agnieszka Guzik, Andżelina Wolan-Nieroda, Mariusz Drużbicki

**Affiliations:** Department of Physiotherapy, Institute of Health Sciences, Medical College, University of Rzeszów, Rzeszów, Poland

**Keywords:** gait analysis, observational gait analysis, 3D gait analysis, hemiparetic gait, stroke, application software

## Abstract

Currently, there are no computerized tools enabling objective interpretation of observational gait assessment based on Wisconsin Gait Scale (WGS), which is a reliable and well-tested tool. The solution envisaged by us may provide a practical tool for assessing gait deviations in patients with hemiparesis after stroke. The present study assessed agreement between a new application software for computerized WGS and 3-dimensional gait analysis (3DGA), and reliability of the application. The study involved 33 individuals with hemiparesis after stroke. The software was developed based on a model designed taking into account components of the WGS and incorporating auxiliary lines passing through the relevant anthropometric points on the patient’s body, as well as measurements of angular values, distances and duration of the specific gait phases, which make it possible to substantiate assessment based on this scale. Series of videos were made to record gait of the qualified patients. After the gait evaluation was carried out using the app, the data were retrieved from the software. The gait assessment was performed separately by three independent examiners who reviewed the video recording using the new app twice (two weeks apart). Additionally, 3DGA was carried out for all the subjects, and the results of the app-aided assessment were compared to those acquired using 3DGA. The findings show statistically significant correlations (*p* < 0.05) between majority of the WGS items measured using the new app, and the relevant spatiotemporal and kinematic parameters identified by 3DGA. Agreement between the scores reported by the three examiners was high in both measurements, as reflected by Cronbach’s alpha exceeding 0.8. The findings reflect very good intra-observer reliability (as reflected by kappa coefficients from 0.847 to 1) and inter-observer reliability (as reflected by kappa coefficients from 0.634 to 1) of the new application software for computerized WGS. The opportunities offered by the observational gait scale objectified through our new software for computerized WGS result from the fact that the tool provides a useful low-cost and time-effective feedback to monitor ongoing treatments or formulate hypotheses.

## Introduction

Given the fact that the ability to walk is crucial for personal autonomy and the quality of life, hemiparetic gait analysis is of great importance from the viewpoint of stroke rehabilitation. During the entire process, clinicians must regularly evaluate improvements in the gait of patients with stroke to assess effectiveness of treatments or therapies (Li et al., 2018; Marin et al., 2020; Sánchez and Winstein, 2021).

Objective 3-dimensional gait analysis (3DGA) is a gold standard in gait assessment since it provides a combination of kinematics, kinetic and electromyography (EMG) data, and as a result is useful in treatment selection and in clinical decision-making. Indeed, instrumental gait analysis and EMG are considered among the fundamental sources of information to drive treatment selection (McGinley et al., 2009; Campanini et al., 2020). However, 3DGA is rather costly, and the related measurements provide a large body of data to be interpreted, which may be inconvenient in the clinical setting. Due to these drawbacks, many researchers worldwide are looking for new solutions specifically enabling gait analysis in patients after stroke (Ferrarello et al., 2013; Begg et al., 2014; Solanki and Lahiri, 2018; Li et al., 2019; Tian et al., 2019; Seo et al., 2020; Iosa et al., 2021; Mohan et al., 2021; Wang et al., 2021). As an example, Seo et al. proposed an assessment method referred to as clinometric gait analysis and applying a smart insoles technology. In a pilot study, they examined twenty-two gait parameters in relation to selected stroke severity data, including scores in Fugl-Meyer Assessment (FMA) related to lower and upper limbs, the Mini–Mental State Examination as well as the Modified Barthel Index. Ten out of the 22 parameters presented significant correlations (difference in stance and swing duration, sound-side stance and swing duration, hemiplegic-side stance duration, single support time, cadence, walking speed). The researchers reported the strongest correlations between the FMA lower-extremity scores and the differences between the unaffected and hemiplegic sides in stance duration (Seo et al., 2020). Generally, however, many of these novel approaches, based on advanced technologies, may present similar practical challenges as far as their everyday use in clinical settings. Due to this, simple and affordable observational methods are still commonly used since they allow clinicians to quickly perform quantitative evaluation of deviations from normal gait patterns (Allen and Neptune, 2012; Gor-García-Fogeda et al., 2016; Estrada-Barranco et al., 2021). On the other hand, observational scales or tests apply subjective methods, naturally associated with a certain margin of error. Furthermore, since there are no consistent standards applicable to observational gait analyses, it is difficult to compare findings reported by various researchers. In view of the above, there is a need for a highly specific and accurate tool enabling both detailed evaluation of visible motor changes and effective monitoring of progress achieved in the course of rehabilitation.

The available tools for visual gait assessment include the simple Observational Gait Analysis Checklist adapted from a checklist developed by the Professional Staff Association of Rancho Los Amigos Medical Center; the former tool is based on a short list of gait deficits and the examiner is required to make yes/no decisions about their presence (Downey, 1989). Scales demonstrating a higher methodological quality and designed for use in assessing patients with central nervous system disorders include the Visual Gait Assessment Scale, Salford Gait Tool, Edinburgh Visual Gait Score (Rathinam et al., 2014; Ridao-Fernández et al., 2019) as well as Gait Assessment and Intervention Tool (Gor-García-Fogeda et al., 2016). Conversely, the Wisconsin Gait Scale (WGS), proposed in 1996 by Rodriquez et al. (1996) was specifically intended for individuals with hemiparesis after stroke. It was designed to enable quick assessment of fourteen visible gait parameters, and consequently to facilitate evaluation of progress achieved in course of rehabilitation programs specifically by patients with hemiparesis after stroke. The factors assessed using WGS include hip, knee and ankle kinematics during specific gait phases (stance and swing), spatiotemporal gait parameters (e.g., length of step, duration of stance phase on the specific sides), symmetry between the affected and unaffected sides of the body, postural balance, as well as the need for orthopedic devices (Rodriquez et al., 1996; Estrada-Barranco et al., 2021). A number of studies have demonstrated reliability and validity of WGS for gait analysis in subjects with hemiparesis after stroke (Rodriquez et al., 1996; Turani et al., 2004; Pizzi et al., 2007; Yaliman et al., 2014; Lu et al., 2015; Wellmon et al., 2015; Guzik et al., 2016; Estrada-Barranco et al., 2019, 2021; Murciano Casas et al., 2020). It has also been shown to correlate well with 3DGA and with tools assessing performance, balance, and independence at different stages of evolution post-stroke (Rodriquez et al., 1996; Turani et al., 2004; Pizzi et al., 2007; Yaliman et al., 2014; Lu et al., 2015; Wellmon et al., 2015; Guzik et al., 2016; Estrada-Barranco et al., 2019, 2021; Murciano Casas et al., 2020). Obviously, in addition to the positive metric properties, the WGS also presents a limitation as regards the final score because to sum up arbitrary ordinal item scores into a total score and treating this score as a number is methodologically wrong (Boateng et al., 2018).

Currently, no computerized tools are available to facilitate the use and objective interpretation of observational gait assessment performed using WGS, which is a reliable and well-tested tool (Rodriquez et al., 1996; Turani et al., 2004; Pizzi et al., 2007; Yaliman et al., 2014; Lu et al., 2015; Wellmon et al., 2015; Guzik et al., 2016; Estrada-Barranco et al., 2019, 2021). The solution envisaged by us may provide a fast, simple and useful tool for assessing gait deviations in patients with hemiparesis after stroke. Hence, the study presents an innovative approach in the area of clinimetrics, since no attempts have previously been made to objectify the descriptive WGS whose high biometric value (psychometric properties) has been demonstrated by a number of studies (Rodriquez et al., 1996; Turani et al., 2004; Pizzi et al., 2007; Yaliman et al., 2014; Lu et al., 2015; Wellmon et al., 2015; Guzik et al., 2016; Estrada-Barranco et al., 2019, 2021; Murciano Casas et al., 2020). The gains of this novel approach are linked with the fact that the new application software, taking into account analysis of a given person’s gait pattern, will create a quantitative representation of gait and will make it possible to record the results using a standardized template. The ultimate objective of the project was to develop a computer-aided observational assessment tool which may be helpful for clinicians in formulating hypotheses on clinical outcomes, and in monitoring the effectiveness of the ongoing rehabilitative treatments for patients with hemiparetic gait.

The present study aimed to test the agreement between the data collected by the new application software for computerized WGS and the data acquired using 3DGA; it also assessed the intrarater and interrater reliability of the data collected by the application.

## Methods

### Sample Size

The minimum sample size for the population investigated was determined using sample size calculator (“PLUS module” from Statistica 13.3 software), taking into account the number of individuals with stroke receiving treatment in the rehabilitation clinic in one year, and with the following parameters specified: a fraction size 0.9, a maximum error of 6%, and a 95% confidence level. Ultimately, a sample size of 31 patients was determined.

The following formula was used for the sample size calculation:


$$N\,\min=\frac{\mathrm{NP}\,\left[\alpha \,2\cdot f\,\left(1-f\right)\right]}{\mathrm{NP}\cdot \mathrm{e2}+\alpha \,2\cdot f\,\left(1-f\right)}$$

*N*min – minimum sample size

NP – size of the population from which the sample is drawn

α – level of confidence for the results

f - fraction size

e – expected maximum error

### Participants

The study group consisted of thirty-three individuals (24 males, 9 females; aged 42–79 years) after a single ischemic stroke, at least 6 months from the incident (46.3 ± 44.14 months post-stroke), diagnosed with post-stroke hemiparesis, and able to walk a distance of 10 meters without assistance of another person. Ambulatory assistive devices were permitted during the trials (see Table 1 for the characteristics of the study participants). Patients unable to follow instructions and those with walking skills impaired due to orthopedic or other neurologic conditions were excluded from the study. The study protocol was reviewed and accepted by the local Bioethics Commission at University of Rzeszow’s Medical Faculty. The study design complied with the Declaration of Helsinki. All the study participants gave their informed consent in writing.

**TABLE 1:** Characteristics of the study participants.

Subject (*N* = 33)	
Gender (females/males), *N*	9/24
Hemiparesis (left/right), *N*	12/21
Age (years), Mean (SD)	60.75 (9.7)
Mass (kg), Mean (SD)	73 (8.3)
Height (m), Mean (SD)	1.71 (0.9)
Time since stroke (months), Mean (SD)	46.3 (44.14)
Gait speed (m/s), Mean (SD)	0.75 (0.24)
Fugl-Meyer (lower limb) score, Mean (SD)	23.4 (3.6)

*N, number of subjects; SD, standard deviation.*

### Measures

**FIGURE 1:**
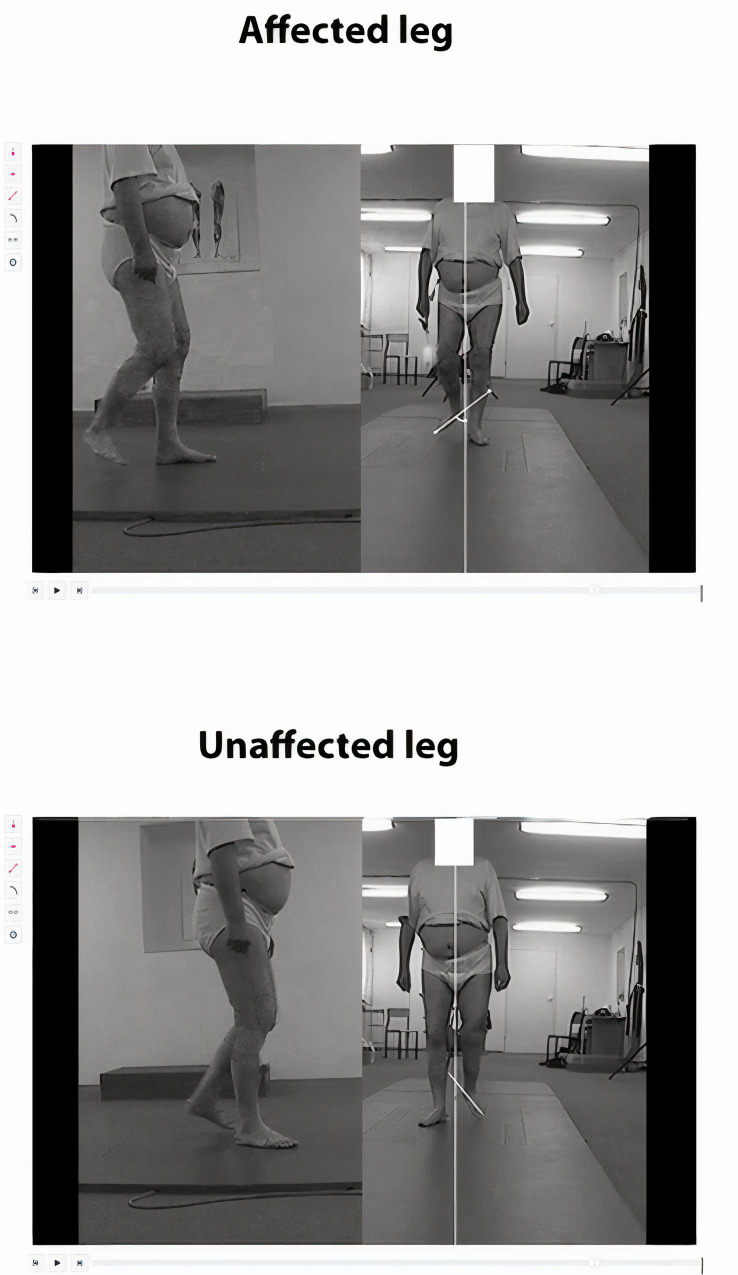
A new application for computerized gait analysis. Subject with right hemiparesis after stroke. Sample assessment of a selected gait parameter in WGS. Swing phase of the affected leg - external rotation during initial swing. On the freeze frame showing the frontal view, we draw two auxiliary straight lines and measure the angle between them. One line shows the direction of gait and runs in the middle of the distance between the medial malleoli, and the other line marks the long axis of the foot (in the middle of the distance between the heads of the first and the fifth metatarsal bones or between the calcaneal tuber and the second toe). The result is compared to the healthy leg. The examiner checks the angles measured and determines the rating (the same angles in both legs, reflecting a normal condition - 1 point; greater angle on the paretic side, but lower than 45 degrees - 2 points; angle exceeding 45 degrees on the paretic side - 3 points).

The newly developed application software, dedicated to WGS, comprises 14 items related to assessment of the specific gait phases: stance phase, toe off, swing phase, and heel strike. All these items are rated on a 3-point scale, except for Item 1 which is rated on a 5-point scale, and Item 11 rated on a 4-point scale. The subjects can obtain a maximum score of 42 points and a minimum score of 13.35, a lower score reflecting higher quality of the gait pattern (Rodriquez et al., 1996). We developed the code, and a system comprising a module for analysis of images, a database making it possible to process the data collected by the image analysis module, as well as a module for reporting the data from the conducted examinations. The software implements the Model-View-Controller (MVC) design pattern and was executed using technologies and solutions that enable running applications in a standard PC environment (web browser). The system uses a relational database as well as object-oriented programming and scripting languages. Data analysis and data processing take place in a dedicated reporting module which makes it possible to export data to an Excel format. The software, based on a developed model utilizing the components of WGS, was additionally provided with auxiliary lines passing through the relevant anthropometric points on the patient’s body, as well as measurements of angular values, distances and duration of the specific gait phases, which make it possible to substantiate assessment based on this scale. The assessments were carried out on selected frames matching specific WGS items (auxiliary lines and angles were marked on the specific freeze frames matched to the WGS items). A detailed description of the method applied in drawing the auxiliary lines and angles for the specific items assessed by the app is presented in Supplementary Table 1. Sample assessment of selected gait parameters are shown in Figure 1.

Series of videos were made to record gait of the patients enrolled in the study. The video recording and the 3D recording were done simultaneously, with two video cameras (BTS Vixta, BTS Bioengineering Corp., Brooklyn, NY, United States) working in synchronicity, and the recording was done in both the sagittal and the frontal plane. The walking distance of 10 meters was defined. A minimum of 6 walks were recorded, including 3 walks involving the affected and 3 walks involving the unaffected side. During the trials the participants walked at a comfortable (self-selected) speed, and were permitted to use their orthopedic aids. After the gait analysis was carried out using the app, the data were retrieved from the software. The gait analysis was performed separately by three independent examiners who reviewed the video recording using the new app twice (two weeks apart). Finally, the results of the app-aided assessment were compared to those obtained using 3DGA.

The 3DGAs were performed with a motion capture system (BTS SMART-DX 700, 250 Hz) comprising two force-plates, six cameras as well as the related software, with SMART Capture, Tracker and Analyzer functions. Passive markers were placed on the participants’ skin, in compliance with Davis Marker Placement system (Davis et al., 1991). During the examination, six or more passages over a distance of 10 meters were recorded for each subject. The participants were instructed to walk at a pace that felt natural to them. Tracker and Analyzer programs (BTS Bioengineering) were applied to compute the mean values of spatiotemporal and kinematic measures based on complete records. Further analyses took into account the following parameters: (1) spatiotemporal: stance time, step length, step width, and (2) kinematic parameters of hip in sagittal, frontal and transverse planes, knee in sagittal plane, and ankle joints as well as pelvis in frontal and transverse planes.

### Data Analysis

The acquired materials were subjected to statistical analyses using Statistica 13.3. Distributions of the investigated variables were examined for normality using Shapiro—Wilk *W*-test. The descriptive statistics computed for the numerical variables (parameters from computerized WSG and 3DGA) included the mean, median, minimum, and maximum values, the first and third quartile, as well as standard deviation.

#### Data Analysis Strategy to Test the Agreement Between Data Collected by the Application for Computerized WSG and Data From 3-Dimensional Gait Analysis

Correlation of two variables failing to meet the criterion of normality of distribution was determined using Spearman’s rank correlation coefficient (0.3 ≤ | *R*| < 0.5 low correlation; 0.5 ≤ | *R*| < 0.7 moderate correlation; 0.7 ≤ | *R*| < 0.9 strong correlation; 0.9 ≤ | *R*| < 1 very strong correlation) (Mukaka, 2012). Analyses were carried out to examine the potential correlations between the matching pairs of variables assessed using the WGS App and 3D Gait Analysis, i.e., we investigated whether the results in the specific items of gait assessment in the WGS App correlated with the results for the corresponding spatiotemporal and kinematic parameters in 3DGA. Statistical significance was assumed if *p* < 0.05. The following matching pairs of variables were defined:

• App stance time on affected side versus 3DGA stance time [s] on affected side (it was assumed that longer 3DGA stance time [s] on affected side was better, corresponding to a lower score (better result) in App stance time on affected side) (Patterson et al., 2008; Mukaino et al., 2016).
• App step length on unaffected side versus 3DGA step length [m] on unaffected side (it was assumed that greater 3DGA step length [m] on unaffected side was better, corresponding to a lower score (better result) in App step length on unaffected side) (Oken and Yavuzer, 2008; Patterson et al., 2008; Lauzière et al., 2014).
• App weight shift to affected side versus 3DGA pelvic oblique range of motion (ROM) on affected side (it was assumed that higher value of 3DGA pelvic oblique range of motion (ROM) on affected side was better, corresponding to a lower score (better result) in App weight shift to affected side)
• App stance width versus 3DGA step width [m] (it was assumed that lower value of 3DGA step width [m] was better, corresponding to a lower score (better result) in App stance width) (Mukaino et al., 2016).
• App guardedness (pause prior to advancing affected leg) versus 3DGA stance time [s] on unaffected side (it was assumed that shorter 3DGA stance time [s] on unaffected side was better, corresponding to a lower score (better result) in App guardedness) (Mukaino et al., 2018).
• App hip extension on affected side versus 3DGA hip flexion/extension (FE) ROM on affected side (it was assumed that higher value of 3DGA hip FE ROM on affected side was better, corresponding to a lower score (better result) in App hip extension on affected side) (Kim and Eng, 2004; Boudarham et al., 2013).
• App external rotation during initial swing on affected side versus 3DGA hip internal/external rotation (IE) ROM on affected side (it was assumed that lower value of 3DGA hip IE ROM on affected side was better, corresponding to a lower score (better result) in App external rotation during initial swing of affected side).
• App circumduction at mid swing on affected side versus 3DGA hip abduction/adduction (AA) ROM on affected side (it was assumed that lower value of 3DGA hip AA ROM on affected side was better, corresponding to a lower score (better result) in App circumduction at mid swing on affected side).
• App hip hiking at mid swing on affected side versus 3DGA hip FE ROM and pelvic oblique ROM on affected side (it was assumed that higher value of 3DGA hip FE ROM on affected side and lower value of 3DGA pelvic oblique ROM on affected side were better, corresponding to a lower score (better result) in App hip hiking at mid swing on affected side) (Mukaino et al., 2018).
• App knee flexion from toe off to mid swing on affected side versus 3DGA knee FE ROM on affected side (it was assumed that higher value of 3DGA knee FE ROM on affected side was better, corresponding to a lower score (better result) in App knee flexion from toe off to mid swing on affected side) (Matsuda et al., 2016; Mukaino et al., 2018; Haruyama et al., 2021).
• App toe clearance on affected side versus 3DGA total between ankle flex in initial contact phase (IC) and ankle flex in toe off phase (TO) on affected side (it was assumed that higher 3DGA total between ankle flex IC and ankle flex TO on affected side was better; patient more effectively controls the foot, and during the swing phase there is a lower risk of toe catch, while the ankle remains in dorsiflexion until it reaches the natural position; this corresponds to a lower score (better result) in App toe clearance on affected side) (Matsuda et al., 2016; Haruyama et al., 2021).
• App pelvic rotation at terminal swing on affected side versus 3DGA pelvic rotation ROM on affected side (it was assumed that higher value of 3DGA pelvic rotation ROM on affected side was better, corresponding to a lower score (better result) in App pelvic rotation at terminal swing on affected side).
• App initial foot contact on affected side versus 3DGA ankle flex IC on affected side (it was assumed that the value of 3DGA ankle flex IC on affected side closer to zero was better, corresponding to a lower score (better result) in App initial foot contact on affected side).

#### The Data Analysis Strategy to Test the Agreement of the Data Collected by the Application for Computerized WSG (Reliability)

The differences in the median levels of a numerical characteristic were assessed using Kruskal—Wallis ANOVA, which was applied to compare the results obtained by the three independent examiners during measurement 1 and measurement 2. On the other hand, Wilcoxon signed-rank test was applied to compare results identified by the same examiner in measurement 1 and measurement 2. Internal reliability of the tool was measured using Cronbach’s alpha. Inter- and intrarater reliability for qualitative variables was assessed with kappa coefficient. Cohen’s version was used for two measurements whereas Fleiss’ version was applied for more than two measurements. Significance level for all statistical tests was set to 0.05.

## Results

Descriptive statistics summarizing the trends in all the measures of computerized WSG and 3DGA are shown in Supplementary Tables 2, 3, respectively (Supplementary Materials).

### Agreement Between the Data Collected by the Application for Computerized WGS and the 3-Dimensional Gait Analysis Data

Statistically significant correlations were identified between majority of the scores in gait assessment carried out using the newly developed application software and the matching spatiotemporal or kinematic parameters measured with 3DGA. Strong correlations (0.7 ≤ | *R*| < 0.9) were found between the following pairs: WGS App external rotation during initial swing affected side versus 3DGA hip IE ROM affected side, as well as WGS App circumduction at mid swing affected side versus 3DGA hip AA ROM affected side. Moderate correlations (0.5 ≤ | *R*| < 0.7) were found in the case of: WGS App step length unaffected side versus 3DGA stride length unaffected side, WGS App hip extension affected side versus 3DGA hip FE ROM affected side, WGS App external rotation during initial swing affected side versus 3DGA hip IE ROM affected side, WGS App circumduction at mid swing affected side versus 3DGA hip AA ROM affected side, WGS App hip hiking at mid swing affected side versus 3DGA hip FE ROM affected side, WGS App knee flexion from toe off to mid swing affected side versus 3DGA knee FE ROM affected side, WGS App toe clearance affected side versus 3DGA total ankle flex IC and ankle flex TO affected side, as well as WGS App initial foot contact affected side versus 3DGA ankle flex IC affected side. Low correlations (0.3 ≤ | *R*| < 0.5) were identified between the pairs: WGS App stance time affected side versus 3DGA stance time affected side, WGS App step length unaffected side versus 3DGA step length [m] unaffected side, WGS App stance width versus 3DGA step width, WGS App hip extension affected side versus 3DGA hip FE ROM affected side, WGS App circumduction at mid swing affected side versus 3DGA hip AA ROM affected side, as well as WGS App pelvic rotation at terminal swing affected side versus 3DGA pelvic rotation ROM affected side. Statistically significant correlations were identified in both assessments performed by all three examiners (Supplementary Table 4).

The findings show no statistically significant correlations only in the case of three pairs of variables: WGS App weight shift to affected side versus 3DGA pelvic oblique ROM affected side, WGS App guardedness versus 3DGA stance time affected side, and WGS App hip hiking at mid swing affected side versus 3DGA pelvic oblique ROM affected side (Supplementary Table 5).

### Agreement of the Data Collected by the Application for Computerized WSG (Reliability)

#### Reliability of Computerized WGS Values as Assessed by the Therapists

The results obtained by the three examiners did not differ significantly in measurements 1 and 2 (Table 2). At the next stage the results obtained by the specific therapists were examined for correlations. It was shown that there were no statistically significant differences between the results obtained in measurement 1 and 2 by the specific examiners.

**TABLE 2:** Comparison of the results obtained by three independent examiners in measurement 1 and measurements 2.

Items of computerized WGS	Comparison of the results obtained by three independent examiners in measurement 1 p	Comparison of the results obtained by three independent examiners in measurement 2 p
STANCE PHASE AFFECTED LEG use of hand-held gait aid	1.000	1.000
stance time on affected side	0.991	0.916
step length on unaffected side	0.656	0.555
weight shift to affected side	0.960	0.943
stance width	0.820	0.994
TOE OFF AFFECTED LEG guardedness (pause prior to advancing affected leg)	0.643	0.633
hip extension on affected side	0.635	0.480
SWING PHASE AFFECTED LEG external rotation during initial swing	0.922	0.864
circumduction at mid swing	0.807	0.689
hip hiking at mid swing	0.970	0.884
knee flexion from toe off to mid swing	1.000	1.000
toe clearance	1.000	1.000
pelvic rotation at terminal swing	1.000	1.000
HEEL STRIKE AFFECTED LEG initial foot contact	1.000	1.000
points – final score	0.970	0.964

*p, probability index in Kruskal—Wallis ANOVA.*

#### Intrarater and Interrater Reliability

Intrarater reliability was measured using Cronbach’s alpha. Agreement between the scores reported by the three examiners was high in both measurements, as reflected by Cronbach’s alpha exceeding 0.8 (Table 3). Inter- and intrarater reliability was assessed with kappa coefficient. Comparative analysis of two measurements performed by the same examiner (intrarater reliability), showed very high and in some cases perfect agreement for all three examiners, as reflected by kappa coefficients ranging from 0.847 to 1 (from 91.92% to 100% agreement) – Table 4. Comparison of measurements performed by three examiners (interrater reliability), in both measurements showed moderate to perfect agreement, as reflected by kappa coefficients from 0.634 to 1 (from 72.73% to 100% agreement) – Table 5.

**TABLE 3:** Agreement between measurements 1 and 2 performed by examiners 1,2 and 3.

Cronbach’s alpha	Examiner 1		Examiner 2		Examiner 3	
	1	2	1	2	1	2
	0.84	0.83	0.81	0.81	0.81	0.81
STANCE PHASE AFFECTED LEG use of handheld gait aid	0.83	0.82	0.80	0.80	0.81	0.80
stance time on affected side	0.83	0.82	0.80	0.80	0.80	0.79
step length on unaffected side	0.82	0.80	0.79	0.80	0.80	0.80
weight shift to affected side	0.86	0.85	0.84	0.83	0.83	0.83
stance width	0.85	0.83	0.81	0.81	0.82	0.81
TOE OFF AFFECTED LEG guardedness (pause prior to advancing affected leg)	0.83	0.82	0.81	0.80	0.81	0.80
hip extension on affected side	0.83	0.81	0.80	0.80	0.80	0.80
SWING PHASE AFFECTED LEG external rotation during initial swing	0.83	0.80	0.79	0.80	0.80	0.79
circumduction at mid swing	0.82	0.80	0.77	0.77	0.80	0.79
hip hiking at mid swing	0.82	0.80	0.79	0.79	0.80	0.79
knee flexion from toe off to mid swing	0.81	0.79	0.77	077	0.78	0.77
toe clearance	0.84	0.82	0.80	0.80	0.81	0.80
pelvic rotation at terminal swing	0.82	0.80	0.77	0.77	0.79	0.78
HEEL STRIKE AFFECTED LEG initial foot contact	0.81	0.79	0.76	0.76	0.78	0.77

**TABLE 4:** Comparison of two measurements performed by the same examiner (intrarater reliability), for all three examiners.

Items of computerized WGS	Kappa coefficient	95% Confidence Interval		Agreement	Interpretation
STANCE PHASE AFFECTED LEG use of hand-held gait aid	1.000	1.000	1.000	100.00%	Perfect
stance time on affected side	0.921	0.847	0.996	95.6%	Nearly perfect
step length on unaffected side	0.902	0.810	0.995	95.6%	Nearly perfect
weight shift to affected side	0.896	0.816	0.977	93.4%	Strong
stance width	0.895	0.806	0.984	94.5%	Strong
TOE OFF AFFECTED LEG guardedness (pause prior to advancing affected leg)	0.847	0.750	0.944	91.2%	Strong
hip extension on affected side	0.898	0.813	0.984	94.5%	Strong
SWING PHASE AFFECTED LEG external rotation during initial swing	0.981	0.945	1.000	98.9%	Nearly perfect
circumduction at mid swing	0.965	0.916	1.000	97.8%	Nearly perfect
hip hiking at mid swing	0.983	0.948	1.000	98.9%	Nearly perfect
knee flexion from toe off to mid swing	1.000	1.000	1.000	100.00%	Perfect
toe clearance	1.000	1.000	1.000	100.00%	Perfect
pelvic rotation at terminal swing	1.000	1.000	1.000	100.00%	Perfect
HEEL STRIKE AFFECTED LEG initial foot contact	1.000	1.000	1.000	100.00%	Perfect

**TABLE 5:** Comparison of measurements performed by three different examiners (interrater reliability), in both examinations.

Items of computerized WGS	Kappa coefficient	95% Confidence Interval		Agreement	Interpretation
STANCE PHASE AFFECTED LEG use of hand-held gait aid	1.000	1.000	1.000	100.00%	Perfect
stance time on affected side	0.902	0.816	0.980	92.42%	Nearly perfect
step length on unaffected side	0.634	0.458	0.783	77.27%	Moderate
weight shift to affected side	0.706	0.578	0.822	74.24%	Moderate
stance width	0.789	0.664	0.895	84.85%	Moderate
TOE OFF AFFECTED LEG guardedness (pause prior to advancing affected leg)	0.655	0.512	0.778	72.73%	Moderate
hip extension on affected side	0.715	0.589	0.845	78.79%	Moderate
SWING PHASE AFFECTED LEG external rotation during initial swing	0.794	0.670	0.900	83.33%	Moderate
circumduction at mid swing	0.859	0.750	0.947	87.88%	Strong
hip hiking at mid swing	0.948	0.878	1.000	95.45%	Nearly perfect
knee flexion from toe off to mid swing	1.000	1.000	1.000	100.00%	Perfect
toe clearance	1.000	1.000	1.000	100.00%	Perfect
pelvic rotation at terminal swing	1.000	1.000	1.000	100.00%	Perfect
HEEL STRIKE AFFECTED LEG initial foot contact	1.000	1.000	1.000	100.00%	Perfect

## Discussion

This study investigated agreement between data collected by a new application software for computerized WGS and 3DGA data, and reliability of the data collected by the application. We initiated the related research since the qualitative and quantitative methods most commonly used to evaluate walking ability in patients, in addition to certain advantages also present serious drawbacks, and due to this there is a need to objectify the descriptive gait analysis.

The results of the app-aided assessment were compared to those obtained using 3DGA. We examined the correlations with fourteen 3D gait parameters, which included spatiotemporal and kinematic measures. We matched the corresponding 3D parameters with the specific items of gait assessment performed using the application software dedicated to the WGS, which is designed to evaluate a variety of factors, including spatial (step length, stance width), temporal (stance time) as well as kinematic (pelvic, hip, knee, ankle ROM) parameters of gait. The latter aspect explains the value of WSG and the advantages it presents over other scales as regards evaluation of gait in individuals with hemiparesis post-stroke (Gor-García-Fogeda et al., 2016; Estrada-Barranco et al., 2019, 2021). Of the fourteen spatiotemporal and kinematic 3D gait parameters, eleven showed significant correlations. This means that both the kinematic measures related to lower limb joints (hip, knee, and ankle) and the spatial measures of gait determined using the newly developed application software are highly consistent with the results of 3DGA, which is the most objective method enabling evaluation of walking ability. This suggests that the proposed application software dedicated to the WGS is a promising tool for gait analysis. We should also point out, however, that Rathinam et al. performed a systematic review of observational gait assessment tools, in terms of their validity and consistency relative to instrumented gait analysis methods. They reported that none of the observational tools were as reliable as the instrumented methods, even though the Edinburgh Visual Gait Score was found to achieve a greater level of consistency, compared to the other tools. The authors also point out that there are very few studies investigating these specific observational tools, hence there is insufficient evidence to determine their clinical validity (Rathinam et al., 2014).

In the present study, no statistically significant correlations were identified only in the case of three matching pairs of variables: WGS App weight shift to affected side versus 3DGA pelvic oblique ROM affected side; WGS App hip hiking at mid swing affected side versus 3DGA pelvic oblique ROM affected side; and WGS App guardedness versus 3DGA stance time affected side. The only 3D kinematic parameter which did not correlate with the matching measure of gait determined using the WGS App was pelvic oblique ROM on affected side. We believe this may be linked to the fact that in order to calculate pelvic oblique ROM in 3DGA in line with Davis Marker Placement system, the passive markers are positioned on the pelvis on the anterior and posterior iliac spine (Davis et al., 1991), which may be problematic in the case of patients after stroke, frequently presenting with obesity. It is very difficult to find the accurate place and to position the marker in the required anthropometric point on the pelvis of a patient with obesity because the marker moves with a skin fold or it is frequently partly covered up by the abdominal walls; this may adversely affect accurate recording of pelvic oblique ROM. On the other hand, the lack of statistically significant correlations between WGS App guardedness (pause prior to advancing affected leg) and 3DGA stance time affected side possibly reflects the fact that matching the former measure with the corresponding 3DGA parameter was problematic. Basically, no 3DGA parameter corresponds to guardedness options described in the WGS as “good forward momentum with no hesitancy noted, or slight/marked hesitation.” Because of this we hypothesized that greater hesitancy or guardedness before advancing affected leg would correspond to longer stance time in seconds. However, the findings do not support this assumption. It is likely that guardedness, i.e., pause prior to advancing affected leg, may be related to a number of factors, such as fear of falling, and due to this we are unable to select one corresponding 3DGA parameter matching the measure of guardedness.

The findings show very good intra- and inter-observer reliability of the new application software. The gait assessment was performed separately by three independent examiners who reviewed the video recording using the new app twice (two weeks apart). Pasqual Marques et al. investigated inter- and intra-observer reliability of hip flexion and abduction measurements performed using computerized photogrammetry and universal goniometer. In that study two independent examiners conducted the measurements twice (one week apart). The authors also concluded that assessments performed using computerized photogrammetry and universal goniometer were highly reliable, while the correlations between measurements of flexion and abduction based on the two methods were either excellent or very good, meaning that both methods were valid (Pasqual Marques et al., 2017). Notably, some free tools available today make it possible to assess some kinematic characteristics, based on good quality videos. One of these is Kinovea, a free open-source tool intended for sport analysis, and making it possible to record, slow down, compare, annotate as well as measure motion in videos. In fact, research has shown that Kinovea software is a valid and reliable tool (Puig-Diví et al., 2019).

In summary, the analyses based on Spearman’s rank correlation coefficient provided evidence that the results of computerized WGS and 3DGA were similar. This could lead us to a conclusion that an examiner using computerized WGS would be able to see and describe gait with similar accuracy to that achieved by a 3D system. Generally, the findings suggest that this is a tool which will make it possible to clarify disputable situations during assessments based on the WGS. In fact, we have aimed to develop a tool which will make it easier to take decisions, and will facilitate interpretation of observations during the rating process performed with the WGS, based on an optical tool. The WGS is the essential component of the tool, whereas the processing and viewing of the video recording enabled by the app is intended to facilitate accurate scoring in that scale. Our study has demonstrated very good intra- and inter-observer reliability of the new application software which means that owing to the support of the app, WGS-based measurements performed twice by three examiners provide highly consistent information. Generally, the tool which we developed was not intended to replace the existing and available instruments but rather it was designed to facilitate and improve objectivity of assessments performed with the reliable and valid WGS (Rodriquez et al., 1996; Turani et al., 2004; Pizzi et al., 2007; Yaliman et al., 2014; Lu et al., 2015; Wellmon et al., 2015; Guzik et al., 2016; Estrada-Barranco et al., 2019, 2021; Murciano Casas et al., 2020). If the ability of the App to identify changes promoted by treatments (sensitivity of the tool) can be demonstrated, the tool may prove to be useful in the daily practice of clinical professionals.

### Limitations

The first limitation is related to the Davis protocol applied in the study, and in particular to assessment of ankle kinematics, since the equinovarus foot deformity is the most common deviation in stroke survivors, but unfortunately this is not measured by the Davis protocol. We applied the Davis Protocol because it is the most commonly used gait analysis procedure (Tenore et al., 2006), however we are aware of the limitations related to that protocol. Furthermore, no control group was included in the study and the simultaneous very good intra- and inter-observer reliability of the new application software has only been confirmed for patients at a chronic phase of recovery after stroke. It would be justified to carry out another study with a similar design in a group of patients at an early phase of recovery post-stroke, in order to evaluate the ability of the proposed software to discriminate between different levels of motor ability/pathology severity. Moreover, it is necessary to carry out further research to investigate the sensitivity of the tool to changes resulting from treatments applied and rehabilitation programs implemented. Another limitation is related to the fact that, in line with the EU regulation 2017/745 the proposed app is a medical device. This means that it requires a certification procedure before being used with patients and before being proposed to other users.

## Conclusion

The findings show very good intra- and inter-observer reliability of the new application software enabling computerized WGS evaluation of gait in individuals with post-stroke hemiparesis. The opportunities offered by the observational gait scale objectified through our new software for computerized WGS result from the fact that the tool provides a useful low-cost and time-effective feedback to monitor ongoing treatments or formulate hypotheses. It is necessary to carry out further research to assess the sensitivity of the tool to changes produced by treatments and rehabilitation programs.

## Data Availability Statement

The raw data supporting the conclusions of this article will be made available by the authors, without undue reservation.

## Ethics Statement

The studies involving human participants were reviewed and approved by Bioethics Committee of the University of Rzeszów. The patients/participants provided their written informed consent to participate in this study.

## Author Contributions

AG: conceptualization, methodology, investigation, formal analysis, and writing—original draft preparation. MD and AW-N: investigation, data curation, and writing—review and editing. All authors contributed to the article and approved the submitted version.

## Conflict of Interest

The authors declare that the research was conducted in the absence of any commercial or financial relationships that could be construed as a potential conflict of interest.

## Publisher’s Note

All claims expressed in this article are solely those of the authors and do not necessarily represent those of their affiliated organizations, or those of the publisher, the editors and the reviewers. Any product that may be evaluated in this article, or claim that may be made by its manufacturer, is not guaranteed or endorsed by the publisher.

## References

[B1] Allen J. L. Neptune R. R. (2012). Three-dimensional modular control of human walking. *J. Biomech.* 45 2157–2163. 10.1016/j.jbiomech.2012.05.037 22727468 PMC3405171

[B2] Begg R. K. Tirosh O. Said C. M. Sparrow W. A. Steinberg N. Levinger P. (2014). Gait training with real-time augmented toe-ground clearance information decreases tripping risk in older adults and a person with chronic stroke. *Front. Hum. Neurosci.* 8:243. 10.3389/fnhum.2014.00243 24847234 PMC4021142

[B3] Boateng G. O. Neilands T. B. Frongillo E. A. Melgar-Quiñonez H. R. Young S. L. (2018). Best Practices for Developing and Validating Scales for Health. *Soc. Behav. Res.* 11:149. 10.3389/fpubh.2018.00149 29942800 PMC6004510

[B4] Boudarham J. Roche N. Pradon D. Bonnyaud C. Bensmail D. Zory R. (2013). Variations in kinematics during clinical gait analysis in stroke patients. *PLoS One* 8:e66421. 10.1371/journal.pone.0066421 23799100 PMC3684591

[B5] Campanini I. Disselhorst-Klug C. Rymer W. Z. Merletti R. (2020). Surface EMG in Clinical Assessment and Neurorehabilitation: barriers Limiting Its Use. *Front. Neurol.* 2:934. 10.3389/fneur.2020.00934 32982942 PMC7492208

[B6] Davis R. B. Õunpuu S. Tyburski D. Gage J. R. (1991). A gait analysis data collection and reduction technique. *Hum. Mov. Sci.* 10 575–587.

[B7] Downey C. A. (1989). *Observational gait analysis handbook. Professional Staff Association.* Washington: Rancho Los Amigos Medical Center Press.

[B8] Estrada-Barranco C. Abuín-Porras V. López-Ruiz J. Sanz-Esteban I. Molina-Rueda F. Cano-de-la-Cuerda R. (2021). Spanish Cross-Cultural Adaptation of the Wisconsin Gait Scale. *Int. J. Environ. Res. Public Health* 18:6903. 10.3390/ijerph18136903 34199082 PMC8297248

[B9] Estrada-Barranco C. Cano-De-La-Cuerda R. Molina-Rueda F. (2019). Construct validity of the Wisconsin Gait Scale in acute, subacute and chronic stroke. *Gait Posture.* 68 363–368. 10.1016/j.gaitpost.2018.12.020 30583192

[B10] Ferrarello F. Bianchi V. A. Baccini M. Rubbieri G. Mossello E. Cavallini M. C. (2013). Tools for observational gait analysis in patients with stroke: a systematic review. *Phys. Ther.* 93 1673–1685. 10.2522/ptj.20120344 23813091

[B11] Gor-García-Fogeda M. D. de la Cuerda R. Tejada M. C. Diego I. M. A. Rueda F. M. (2016). Observational gait as-sessments in people with neurological disorders: a systematic review. *Arch. Phys. Med. Rehabil.* 97 131–140. 10.1016/j.apmr.2015.07.018 26254954

[B12] Guzik A. Drużbicki M. Przysada G. Kwolek A. Brzozowska-Magoń A. Wyszyńska J. (2016). Assessment of test-retest reliability and internal consistency of the Wisconsin Gait Scale in hemiparetic post-stroke patients. *Postep. Rehabil.* 30 41–53. 10.1515/rehab-2015-0048

[B13] Haruyama K. Kawakami M. Okada K. Okuyama K. Tsuzuki K. Liu M. (2021). Pelvis-Toe Distance: 3-Dimensional Gait Characteristics of Functional Limb Shortening in Hemiparetic Stroke. *Sensors* 21:5417. 10.3390/s21165417 34450859 PMC8401521

[B14] Iosa M. Capodaglio E. Pelà S. Persechino B. Morone G. Antonucci G. (2021). Artificial Neural Network analyzing wearable device gait data for identifying patients with stroke unable to return to work. *Front. Neurol.* 12:561. 10.3389/fneur.2021.650542 34093396 PMC8170310

[B15] Kim C. M. Eng J. J. (2004). Magnitude and pattern of 3D kinematic and kinetic gait profiles in persons with stroke: relationship to walking speed. *Gait Posture* 20 140–146. 10.1016/j.gaitpost.2003.07.002 15336283 PMC3167865

[B16] Lauzière S. Betschart M. Aissaoui R. Nadeau S. (2014). Understanding spatial and temporal gait asymmetries in individuals post stroke. *Int. J. Phys. Med. Rehabil.* 2:3. 10.4172/2329-9096.1000201

[B17] Li M. Tian S. Sun L. Chen X. (2019). Gait Analysis for Post-Stroke Hemiparetic Patient by Multi-Features Fusion Method. *Sensors* 19:1737. 10.3390/s19071737 30978981 PMC6479843

[B18] Li S. Francisco G. E. Zhou P. (2018). Post-stroke Hemiplegic Gait: new Perspective and Insights. *Front. Physiol.* 9:1021. 10.3389/fphys.2018.01021 30127749 PMC6088193

[B19] Lu X. Hu N. Deng S. Li J. Qi S. Bi S. (2015). The reliability, validity and correlation of two observational gait scales assessed by video tape for Chinese subjects with hemiplegia. *J. Phys. Ther. Sci.* 27 3717–3721. 10.1589/jpts.27.3717 26834338 PMC4713777

[B20] Marin J. Marin J. J. Blanco T. de la Torre J. Salcedo I. Martitegui E. (2020). Is My Patient Improving? Individualized Gait Analysis in Rehabilitation. *Appl. Sci.* 10:8558. 10.3390/app10238558

[B21] Matsuda F. Mukaino M. Ohtsuka K. Tanikawa H. Tsuchiyama K. Teranishi T. (2016). Biomechanical factors behind toe clearance during the swing phase in hemiparetic patients. *Top Stroke Rehabil.* 24 177–182. 10.1080/10749357.2016.1234192 27671158

[B22] McGinley J. L. Baker R. Wolfe R. Morris M. E. (2009). The reliability of three-dimensional kinematic gait measurements: a systematic review. *Gait Posture.* 29 360–369. 10.1016/j.gaitpost.2008.09.003 19013070

[B23] Mohan D. M. Khandoker A. H. Wasti S. A. Ismail S. Ibrahim Ismail Alali S. (2021). Assessment Methods of Post-stroke Gait: a Scoping Review of Technology-Driven Approaches to Gait Characterization and Analysis. *Front. Neurol.* 12:650024. 10.3389/fneur.2021.650024 34168608 PMC8217618

[B24] Mukaino M. Ohtsuka K. Tanikawa H. Matsuda F. Yamada J. Itoh N. (2018). Clinical-oriented Three-dimensional Gait Analysis Method for Evaluating Gait Disorder. *J. Vis. Exp.* 133:57063. 10.3791/57063 29553535 PMC5931438

[B25] Mukaino M. Ohtsuka K. Tsuchiyama K. Matsuda F. Inagaki K. Yamada J. (2016). Feasibility of a Simplified, Clinically Oriented, Three-dimensional Gait Analysis System for the Gait Evaluation of Stroke Patients. *Prog. Rehabil. Med.* 15:20160001. 10.2490/prm.20160001 32789198 PMC7372005

[B26] Mukaka M. M. (2012). A guide to appropriate use of Correlation coefficient in medical research. *Malawi Med. J.* 24 69–71. 23638278 PMC3576830

[B27] Murciano Casas M. P. Zarco Periñán M. J. Corral López I. Álamo Vera V. Ferrand Ferri P. Barrera Chacón J. M. (2020). Development of the Spanish version of the Wisconsin Gait Scale. Reliability and consistency analysis of spatial and temporal parameters with gait assessment in stroke patients. *Rehabilitacion* 10.1016/j.rh.2020.10.003 [Epub Online ahead of print] 33246641

[B28] Oken O. Yavuzer G. (2008). Spatio-temporal and kinematic asymmetry ratio in subgroups of patients with stroke. *Eur. J. Phys. Rehabil. Med.* 44 127–132. 18418332

[B29] Pasqual Marques A. Oshima Marcolan J. N. Nucci Nogueira Prado J. Nogueira Burke T. Alves Gonçalves (2017). Inter- and intra-rater reliability of computerized photogrammetry and universal goniometer in the measurement of hip flexion and abduction. *Fisioter Pesqui.* 24 22–28. 10.1590/1809-2950/15886624012017

[B30] Patterson K. K. Parafianowicz I. Danells C. J. Closson V. Verrier M. C. Staines W. R. (2008). Gait asymmetry in community-ambulating stroke survivors. *Arch. Phys. Med. Rehab.* 89 304–310. 10.1016/j.apmr.2007.08.142 18226655

[B31] Pizzi A. Carlucci G. Falsini C. Lunghi F. Verdesca S. Grippo A. (2007). Gait in hemiplegia: evaluation of clinical features with the wisconsin gait scale. *Acta. Derm. Venereol.* 39 170–174. 10.2340/16501977-0026 17351701

[B32] Puig-Diví A. Escalona-Marfil C. Padullés-Riu J. M. Busquets A. Padullés-Chando X. Marcos-Ruiz D. (2019). Validity and reliability of the Kinovea program in obtaining angles and distances using coordinates in 4 perspectives. *PLoS One.* 14:e0216448. 10.1371/journal.pone.0216448 31166989 PMC6550386

[B33] Rathinam C. Bateman A. Peirson J. Skinner J. (2014). Observational gait assessment tools in paediatrics–a systematic review. *Gait Posture* 40 279–285. 10.1016/j.gaitpost.2014.04.187 24798609

[B34] Ridao-Fernández C. Pinero-Pinto E. Chamorro-Moriana G. (2019). Observational Gait Assessment Scales in Patients with Walking Disorders: systematic Review. *Biomed. Res. Int.* 31:2085039. 10.1155/2019/2085039 31781597 PMC6875351

[B35] Rodriquez A. A. Black P. O. Kile K. A. Sherman J. Stellberg B. McCormnick J. (1996). Gait training efficacy using a home-based practice model in chronic hemiplegia. *Arch. Phys. Med. Rehabil.* 77 801–805. 8702375 10.1016/s0003-9993(96)90260-9

[B36] Sánchez N. Winstein C. J. (2021). Lost in Translation: simple Steps in Experimental Design of Neurorehabilitation-Based Research Interventions to Promote Motor Recovery Post-Stroke. *Front. Hum. Neurosci.* 15:644335. 10.3389/fnhum.2021.644335 33958994 PMC8093777

[B37] Seo M. Shin M. J. Park T. S. Park J. H. (2020). Clinometric Gait Analysis Using Smart Insoles in Patients With Hemiplegia After Stroke: pilot Study. *JMIR mHealth uHealth* 8:e22208. 10.2196/22208 32909949 PMC7516684

[B38] Solanki D. Lahiri U. (2018). Design of instrumented shoes for gait characterization: a usability study with healthy and post-stroke hemiplegic individuals. *Front. Neurosci.* 12:459. 10.3389/fnins.2018.00459 30079008 PMC6062939

[B39] Tenore N. Fortugno F. Viola F. Galli M. Giaquinto S. (2006). Gait analysis as a reliable tool for rehabilitation of chronic hemiplegic patients. *Clin. Exp. Hypertens.* 28 349–355. 10.1080/10641960600549504 16833045

[B40] Tian S. Li M. Wang Y. Chen X. (2019). Application of an Improved Correlation Method in Electrostatic Gait Recognition of Hemiparetic Patients. *Sensors* 19:2529. 10.3390/s19112529 31163585 PMC6603782

[B41] Turani N. Kemiksizog A. Karatas M. (2004). Assessment of hemiplegic gait using the Wisconsin Gait Scale. *Scand. J. Caring. Sci.* 18 103–108. 10.1111/j.1471 15005669

[B42] Wang F. C. Chen S. F. Lin C. H. Shih C. J. Lin A. C. Yuan W. (2021). Detection and Classification of Stroke Gaits by Deep Neural Networks Employing Inertial Measurement Units. *Sensors* 21:1864. 10.3390/s21051864 PMC7962128 33800061

[B43] Wellmon R. Degano A. Rubertone J. A. Campbell S. Russo K. A. (2015). Interrater and intrarater reliability and minimal detectable change of the Wisconsin Gait Scale when used to examine videotaped gait in individuals post-stroke. *Arch. Phys.* 5:11. 10.1186/s40945-015-0011-z 29340180 PMC5759902

[B44] Yaliman A. Kesiktas N. Ozkaya M. Eskiyurt N. Erkan O. Yilmaz E. (2014). Evaluation of intrarater and interrater reliability of the Wisconsin Gait Scale with using the video taped stroke patients in a Turkish sample. *Neurorehabilitation* 34 253–258. 10.3233/NRE-131033 24401828

