# Supplementary material for: Assessment of Agreement Between a New Application to Compute the Wisconsin Gait Score and 3-Dimensional Gait Analysis, and Reliability of the Application in Stroke Patients
Source: Front Hum Neurosci. 2022 Feb 3;16:775261. doi: 10.3389/fnhum.2022.775261 (PMC8851887; doi:10.3389/fnhum.2022.775261)
Supplement: Supplementary file 1 [file Data_Sheet_1.doc]

Supplementary Material

# Supplementary Tables

**Supplementary Table 1.** Detailed description of the method used for determining the auxiliary lines and angles corresponding to the specific items assessed with the app.

| **Computerized WGS items** | **Description/method** |
| --- | --- |
| STANCE PHASE AFFECTED LEG  use of hand-held gait aid | No additional measurements. Examiner performs the rating according to the WGS, based on the video recording. |
| stance time on affected side | Two times (start-stop) are measured on the video – for the step made with affected and unaffected leg. The results for the two legs are then compared. Stance time on affected side is measured from the moment the unaffected leg leaves the ground to the moment the same (unaffected) leg touches the ground. Stance time on unaffected side is measured from the moment the affected leg leaves the ground to the moment the same (affected) leg touches the ground. |
| step length on unaffected side | In a freeze frame with a side view, we mark an auxiliary vertical line corresponding to the position of big toe of the affected leg. We determine whether the heel of the unaffected leg is further than the line corresponding to the big toe of the affected leg. |
| weight shift to the affected side | In a freeze frame with a back view, we draw an auxiliary vertical line running through external occipital protuberance (reflecting the vertical position projected by the torso and the head). In a frontal view we draw an auxiliary vertical line in the middle of the distance between the medial malleoli in the right and the left leg (reflecting the projection point for the centre of gravity of the body). We determine whether the head and the torso move to the side relative to the auxiliary line, i.e., to a position above the foot of the affected leg during a single stance. |
| stance width | Two segments are measured on a freeze frame showing a frontal view. The first one is the foot width (auxiliary “slanting” line connecting metatarsal bones 1 and 5 of the affected leg – corresponding to one-shoe width). The second segment is defined as the distance between the medial malleoli in both legs during the stance phase on affected side. Examiner assesses the difference in these distances and determines whether during stance phase on affected side the distance between the medial malleoli is not greater than one-shoe width, or is not greater than two-shoes width, or exceeds two-shoes width. |
| TOE OFF AFFECTED LEG  guardedness (pause prior to advancing affected leg) | We measure the time (start-stop) on the video. We assess the duration of double support phase (when heel of one leg and toes of the other leg are in contact with the ground), first with the affected leg behind and the unaffected leg in front, and then the other way. If the individual hesitates, the duration of the double support phase will be longer. Greater asymmetry in these two measures (for the two legs) will reflect greater guardedness. |
| hip extension on affected side | On a freeze frame showing a side view, we draw two straight auxiliary lines and we measure the angle between them. One line runs from the hip along the torso up to the armpit, and the other one goes from the hip at the level of the trochanter of the femur down to the knee. The angle between the torso line and the hip-knee line is measured, and compared to the result identified for the unaffected leg. Examiner reviews the values of the angles, and determines the rating. |
| SWING PHASE AFFECTED LEG  external rotation during initial swing | On a freeze frame showing a frontal view, we draw two auxiliary straight lines and measure the angle between them. One line shows the direction of gait and runs in the middle of the distance between the medial malleoli, and the other line marks the long axis of the foot (in the middle of the distance between the heads of the first and the fifth metatarsal bones or between the calcaneal tuber and the second toe). The result is compared to the unaffected leg. Examiner checks the angles measured and determines the rating. |
| circumduction at mid swing | Two segments are measured on a freeze frame with a frontal view. The first one is the foot width (auxiliary “slanting” line connecting metatarsal bones 1 and 5 of the affected leg, corresponding to one-shoe width). The second segment is defined as the distance between the medial malleoli during the swing phase on affected leg. Examiner assesses the difference in the distances and determines whether during swing phase on affected side the distance between the medial malleoli is not greater than one-shoe width, or is not greater than two-shoes width, or exceeds the two-shoes width. |
| hip hiking at mid swing | On a freeze frame showing a frontal view, we draw two auxiliary horizontal lines; one goes through anterior superior iliac spine of the affected leg (situated lower) and the other one, slanting, from the same point through anterior superior iliac spine of the unaffected leg (situated higher). The angle between these two lines is measured, and compared to the result identified for the unaffected leg. Examiner reviews the values of the angles, and determines the rating. |
| knee flexion from toe off to mid swing | On a freeze frame showing a side view, we draw two straight auxiliary lines: along the thigh (trochanter of the femur and lateral condyle of femur), and along the shank (head of the fibula and lateral malleolus). The angle between these two is measured, and compared to the result identified for the unaffected leg. Examiner reviews the values of the angles, and determines the rating. |
| toe clearance | No additional measurements. Examiner performs the rating according to the WGS, based on the video recording. |
| pelvic rotation at terminal swing | On a freeze frame showing a side view, we identify the point on the pelvis with the highest location on the ilium, and we draw an auxiliary vertical line passing through that point. On the same freeze frame, we mark a point on the edge of the buttock and we draw the second vertical line passing through this point. We measure the distance between these lines, and compare the value with the result identified for the unaffected leg. |
| HEEL STRIKE AFFECTED LEG  initial foot contact | No additional measurements. Examiner performs the rating according to the WGS, based on the video recording. |

**Supplementary Table 2. Descriptive statistics for computerized WGS scores**

| Basic descriptive statistics | | | | | | | | |
| --- | --- | --- | --- | --- | --- | --- | --- | --- |
| Parameter | Examiner/measurement | Mean | Median | Min. | Max. | Quartile 1 | Quartile 3 | Standard deviation |
| STANCE PHASE AFFECTED LEG  use of hand-held gait aid | Examiner 1 measurement 1 | 0.78 | 0.60 | 0.60 | 2.40 | 0.60 | 0.60 | 0.51 |
|  | Examiner 1 measurement 2 | 0.78 | 0.60 | 0.60 | 2.40 | 0.60 | 0.60 | 0.51 |
|  | Examiner 2 measurement 1 | 0.78 | 0.60 | 0.60 | 2.40 | 0.60 | 0.60 | 0.51 |
|  | Examiner 2 measurement 2 | 0.78 | 0.60 | 0.60 | 2.40 | 0.60 | 0.60 | 0.51 |
|  | Examiner 3 measurement 1 | 0.78 | 0.60 | 0.60 | 2.40 | 0.60 | 0.60 | 0.51 |
|  | Examiner 3 measurement 2 | 0.78 | 0.60 | 0.60 | 2.40 | 0.60 | 0.60 | 0.51 |
| stance time on affected  side | Examiner 1 measurement 1 | 1.52 | 1.00 | 1.00 | 3.00 | 1.00 | 2.00 | 0.57 |
|  | Examiner 1 measurement 2 | 1.48 | 1.00 | 1.00 | 2.00 | 1.00 | 2.00 | 0.51 |
|  | Examiner 2 measurement 1 | 1.48 | 1.00 | 1.00 | 2.00 | 1.00 | 2.00 | 0.51 |
|  | Examiner 2 measurement 2 | 1.48 | 1.00 | 1.00 | 2.00 | 1.00 | 2.00 | 0.51 |
|  | Examiner 3 measurement 1 | 1.52 | 1.00 | 1.00 | 3.00 | 1.00 | 2.00 | 0.57 |
|  | Examiner 3 measurement 2 | 1.55 | 2.00 | 1.00 | 3.00 | 1.00 | 2.00 | 0.56 |
| step length on unaffected side | Examiner 1 measurement 1 | 1.24 | 1.00 | 1.00 | 3.00 | 1.00 | 1.00 | 0.50 |
|  | Examiner 1 measurement 2 | 1.24 | 1.00 | 1.00 | 3.00 | 1.00 | 1.00 | 0.50 |
|  | Examiner 2 measurement 1 | 1.33 | 1.00 | 1.00 | 3.00 | 1.00 | 2.00 | 0.54 |
|  | Examiner 2 measurement 2 | 1.30 | 1.00 | 1.00 | 2.00 | 1.00 | 2.00 | 0.47 |
|  | Examiner 3 measurement 1 | 1.33 | 1.00 | 1.00 | 3.00 | 1.00 | 2.00 | 0.54 |
|  | Examiner 3 measurement 2 | 1.36 | 1.00 | 1.00 | 3.00 | 1.00 | 2.00 | 0.55 |
| weight shift to affected side | Examiner 1 measurement 1 | 1.73 | 2.00 | 1.00 | 3.00 | 1.00 | 2.00 | 0.72 |
|  | Examiner 1 measurement 2 | 1.70 | 2.00 | 1.00 | 3.00 | 1.00 | 2.00 | 0.73 |
|  | Examiner 2 measurement 1 | 1.67 | 2.00 | 1.00 | 3.00 | 1.00 | 2.00 | 0.65 |
|  | Examiner 2 measurement 2 | 1.61 | 2.00 | 1.00 | 3.00 | 1.00 | 2.00 | 0.56 |
|  | Examiner 3 measurement 1 | 1.70 | 2.00 | 1.00 | 3.00 | 1.00 | 2.00 | 0.64 |
|  | Examiner 3 measurement 2 | 1.67 | 2.00 | 1.00 | 3.00 | 1.00 | 2.00 | 0.65 |
| stance width | Examiner 1 measurement 1 | 1.39 | 1.00 | 1.00 | 3.00 | 1.00 | 2.00 | 0.56 |
|  | Examiner 1 measurement 2 | 1.36 | 1.00 | 1.00 | 2.00 | 1.00 | 2.00 | 0.49 |
|  | Examiner 2 measurement 1 | 1.36 | 1.00 | 1.00 | 2.00 | 1.00 | 2.00 | 0.49 |
|  | Examiner 2 measurement 2 | 1.36 | 1.00 | 1.00 | 2.00 | 1.00 | 2.00 | 0.49 |
|  | Examiner 3 measurement 1 | 1.45 | 1.00 | 1.00 | 3.00 | 1.00 | 2.00 | 0.56 |
|  | Examiner 3 measurement 2 | 1.39 | 1.00 | 1.00 | 3.00 | 1.00 | 2.00 | 0.56 |
| TOE OFF AFFECTED LEG  guardedness (pause prior to advancing affected leg) | Examiner 1 measurement 1 | 1.42 | 1.00 | 1.00 | 3.00 | 1.00 | 2.00 | 0.66 |
|  | Examiner 1 measurement 2 | 1.45 | 1.00 | 1.00 | 3.00 | 1.00 | 2.00 | 0.62 |
|  | Examiner 2 measurement 1 | 1.52 | 1.00 | 1.00 | 3.00 | 1.00 | 2.00 | 0.57 |
|  | Examiner 2 measurement 2 | 1.55 | 2.00 | 1.00 | 3.00 | 1.00 | 2.00 | 0.56 |
|  | Examiner 3 measurement 1 | 1.48 | 1.00 | 1.00 | 3.00 | 1.00 | 2.00 | 0.67 |
|  | Examiner 3 measurement 2 | 1.42 | 1.00 | 1.00 | 2.00 | 1.00 | 2.00 | 0.50 |
| hip extension on affected side | Examiner 1 measurement 1 | 1.42 | 1.00 | 1.00 | 3.00 | 1.00 | 2.00 | 0.66 |
|  | Examiner 1 measurement 2 | 1.42 | 1.00 | 1.00 | 3.00 | 1.00 | 2.00 | 0.66 |
|  | Examiner 2 measurement 1 | 1.36 | 1.00 | 1.00 | 3.00 | 1.00 | 2.00 | 0.60 |
|  | Examiner 2 measurement 2 | 1.33 | 1.00 | 1.00 | 3.00 | 1.00 | 2.00 | 0.54 |
|  | Examiner 3 measurement 1 | 1.48 | 1.00 | 1.00 | 3.00 | 1.00 | 2.00 | 0.62 |
|  | Examiner 3 measurement 2 | 1.48 | 1.00 | 1.00 | 3.00 | 1.00 | 2.00 | 0.57 |
| SWING PHASE AFFECTED LEG  external rotation during initial swing | Examiner 1 measurement 1 | 1.73 | 2.00 | 1.00 | 3.00 | 1.00 | 2.00 | 0.63 |
|  | Examiner 1 measurement 2 | 1.73 | 2.00 | 1.00 | 3.00 | 1.00 | 2.00 | 0.63 |
|  | Examiner 2 measurement 1 | 1.67 | 2.00 | 1.00 | 3.00 | 1.00 | 2.00 | 0.54 |
|  | Examiner 2 measurement 2 | 1.64 | 2.00 | 1.00 | 3.00 | 1.00 | 2.00 | 0.55 |
|  | Examiner 3 measurement 1 | 1.67 | 2.00 | 1.00 | 3.00 | 1.00 | 2.00 | 0.60 |
|  | Examiner 3 measurement 2 | 1.67 | 2.00 | 1.00 | 3.00 | 1.00 | 2.00 | 0.60 |
| circumduction at mid swing | Examiner 1 measurement 1 | 1.61 | 2.00 | 1.00 | 3.00 | 1.00 | 2.00 | 0.66 |
|  | Examiner 1 measurement 2 | 1.58 | 1.00 | 1.00 | 3.00 | 1.00 | 2.00 | 0.66 |
|  | Examiner 2 measurement 1 | 1.64 | 2.00 | 1.00 | 3.00 | 1.00 | 2.00 | 0.65 |
|  | Examiner 2 measurement 2 | 1.64 | 2.00 | 1.00 | 3.00 | 1.00 | 2.00 | 0.65 |
|  | Examiner 3 measurement 1 | 1.55 | 1.00 | 1.00 | 3.00 | 1.00 | 2.00 | 0.67 |
|  | Examiner 3 measurement 2 | 1.52 | 1.00 | 1.00 | 3.00 | 1.00 | 2.00 | 0.67 |
| hip hiking at mid swing | Examiner 1 measurement 1 | 1.61 | 2.00 | 1.00 | 3.00 | 1.00 | 2.00 | 0.66 |
|  | Examiner 1 measurement 2 | 1.61 | 2.00 | 1.00 | 3.00 | 1.00 | 2.00 | 0.66 |
|  | Examiner 2 measurement 1 | 1.61 | 2.00 | 1.00 | 3.00 | 1.00 | 2.00 | 0.66 |
|  | Examiner 2 measurement 2 | 1.61 | 2.00 | 1.00 | 3.00 | 1.00 | 2.00 | 0.66 |
|  | Examiner 3 measurement 1 | 1.64 | 2.00 | 1.00 | 3.00 | 1.00 | 2.00 | 0.65 |
|  | Examiner 3 measurement 2 | 1.67 | 2.00 | 1.00 | 3.00 | 1.00 | 2.00 | 0.65 |
| knee flexion from toe off to mid swing | Examiner 1 measurement 1 | 1.52 | 1.50 | 0.75 | 2.25 | 0.75 | 2.25 | 0.61 |
|  | Examiner 1 measurement 2 | 1.52 | 1.50 | 0.75 | 2.25 | 0.75 | 2.25 | 0.61 |
|  | Examiner 2 measurement 1 | 1.52 | 1.50 | 0.75 | 2.25 | 0.75 | 2.25 | 0.61 |
|  | Examiner 2 measurement 2 | 1.52 | 1.50 | 0.75 | 2.25 | 0.75 | 2.25 | 0.61 |
|  | Examiner 3 measurement 1 | 1.52 | 1.50 | 0.75 | 2.25 | 0.75 | 2.25 | 0.61 |
|  | Examiner 3 measurement 2 | 1.52 | 1.50 | 0.75 | 2.25 | 0.75 | 2.25 | 0.61 |
| toe clearance | Examiner 1 measurement 1 | 1.09 | 1.00 | 1.00 | 2.00 | 1.00 | 1.00 | 0.29 |
|  | Examiner 1 measurement 2 | 1.09 | 1.00 | 1.00 | 2.00 | 1.00 | 1.00 | 0.29 |
|  | Examiner 2 measurement 1 | 1.09 | 1.00 | 1.00 | 2.00 | 1.00 | 1.00 | 0.29 |
|  | Examiner 2 measurement 2 | 1.09 | 1.00 | 1.00 | 2.00 | 1.00 | 1.00 | 0.29 |
|  | Examiner 3 measurement 1 | 1.09 | 1.00 | 1.00 | 2.00 | 1.00 | 1.00 | 0.29 |
|  | Examiner 3 measurement 2 | 1.09 | 1.00 | 1.00 | 2.00 | 1.00 | 1.00 | 0.29 |
| pelvic rotation at terminal swing | Examiner 1 measurement 1 | 1.67 | 1.00 | 1.00 | 3.00 | 1.00 | 3.00 | 0.89 |
|  | Examiner 1 measurement 2 | 1.67 | 1.00 | 1.00 | 3.00 | 1.00 | 3.00 | 0.89 |
|  | Examiner 2 measurement 1 | 1.67 | 1.00 | 1.00 | 3.00 | 1.00 | 3.00 | 0.89 |
|  | Examiner 2 measurement 2 | 1.67 | 1.00 | 1.00 | 3.00 | 1.00 | 3.00 | 0.89 |
|  | Examiner 3 measurement 1 | 1.67 | 1.00 | 1.00 | 3.00 | 1.00 | 3.00 | 0.89 |
|  | Examiner 3 measurement 2 | 1.67 | 1.00 | 1.00 | 3.00 | 1.00 | 3.00 | 0.89 |
| HEEL STRIKE AFFECTED LEG  initial foot contact | Examiner 1 measurement 1 | 1.70 | 2.00 | 1.00 | 3.00 | 1.00 | 2.00 | 0.73 |
|  | Examiner 1 measurement 2 | 1.70 | 2.00 | 1.00 | 3.00 | 1.00 | 2.00 | 0.73 |
|  | Examiner 2 measurement 1 | 1.70 | 2.00 | 1.00 | 3.00 | 1.00 | 2.00 | 0.73 |
|  | Examiner 2 measurement 2 | 1.70 | 2.00 | 1.00 | 3.00 | 1.00 | 2.00 | 0.73 |
|  | Examiner 3 measurement 1 | 1.70 | 2.00 | 1.00 | 3.00 | 1.00 | 2.00 | 0.73 |
|  | Examiner 3 measurement 2 | 1.70 | 2.00 | 1.00 | 3.00 | 1.00 | 2.00 | 0.73 |
| points – final score | Examiner 1 measurement 1 | 20.43 | 19.35 | 13.35 | 32.65 | 16.55 | 23.10 | 5.03 |
|  | Examiner 1 measurement 2 | 20.33 | 19.10 | 13.35 | 30.65 | 16.55 | 23.10 | 4.78 |
|  | Examiner 2 measurement 1 | 20.40 | 19.85 | 14.35 | 29.65 | 16.35 | 23.85 | 4.50 |
|  | Examiner 2 measurement 2 | 20.27 | 20.10 | 14.35 | 29.65 | 16.35 | 23.85 | 4.38 |
|  | Examiner 3 measurement 1 | 20.58 | 20.10 | 13.35 | 32.65 | 16.35 | 23.10 | 4.71 |
|  | Examiner 3 measurement 2 | 20.49 | 20.10 | 13.35 | 32.65 | 17.10 | 22.10 | 4.54 |

**Supplementary Table 3.** Descriptive statistics for 3D analysis

|  | Basic descriptive statistics | | | | | | | |
| --- | --- | --- | --- | --- | --- | --- | --- | --- |
|  | Number | Mean | Median | Min. | Max. | Quartile 1 | Quartile 3 | Standard deviation |
| Stance Time [s] on affected side | 33 | 1.12 | 1.04 | 0.65 | 1.90 | 0.88 | 1.40 | 0.34 |
| Stance Time [s] on unaffected side | 33 |  |  |  |  |  |  |  |
| Stride Time [s] on unaffected side | 33 | 1.72 | 1.55 | 1.18 | 2.61 | 1.31 | 2.17 | 0.47 |
| Stride length [m] on unaffected side | 33 | 0.61 | 0.55 | 0.20 | 1.08 | 0.41 | 0.81 | 0.25 |
| Step Length [m] on unaffected side | 33 | 0.24 | 0.23 | 0.06 | 0.48 | 0.15 | 0.30 | 0.11 |
| Pelvic Obl ROM on affected side | 33 | 6.02 | 5.60 | 1.80 | 13.40 | 4.20 | 7.30 | 2.64 |
| Step Width [m] | 33 | 0.28 | 0.28 | 0.17 | 0.38 | 0.24 | 0.33 | 0.06 |
| Hip FE ROM on affected side | 33 | 25.63 | 25.90 | 10.90 | 42.90 | 21.10 | 31.30 | 7.27 |
| Hip IE ROM on affected side | 33 | 10.49 | 10.00 | 3.10 | 20.50 | 7.80 | 12.80 | 4.04 |
| Hip AA ROM on affected side | 33 | 6.56 | 6.50 | 3.70 | 10.40 | 4.70 | 8.00 | 2.16 |
| Knee FE ROM on affected side | 33 | 35.90 | 35.30 | 10.70 | 56.20 | 30.00 | 46.80 | 12.91 |
| Total between Ankle Flex IC and Ankle Flex TO on affected side | 33 | 16.41 | 15.50 | 0.50 | 39.60 | 9.10 | 22.80 | 9.94 |
| Pelvic Rot ROM on affected side | 33 | 9.96 | 8.60 | 5.90 | 19.10 | 7.00 | 11.60 | 3.78 |
| Ankle Flex IC on affected side | 33 | -2.79 | -4.10 | -16.50 | 18.70 | -12.80 | 7.20 | 9.99 |

**Supplementary Table 4.** Comparison of the results of the app-aided analysis and 3DGA

| **Variables** | **R p** | | |
| --- | --- | --- | --- |
| WGS App stance time affected side versus 3DGA stance time [s] affected side | | | |
| Examiner 1 measurement 1 | | -0.42 | 0.019 |
| Examiner 1 measurement 2 | | -0.31 | 0.025 |
| Examiner 2 measurement 1 | | -0.31 | 0.025 |
| Examiner 2 measurement 2 | | -0.31 | 0.025 |
| Examiner 3 measurement 1 | | -0.42 | 0.019 |
| Examiner 3 measurement 2 | | -0.45 | 0.008 |
| WGS App step length unaffected side versus 3DGA step length [m] unaffected side | | | |
| Examiner 1 measurement 1 | | -0.45 | 0.008 |
| Examiner 1 measurement 2 | | -0.45 | 0.008 |
| Examiner 2 measurement 1 | | -0.62 | <0.001 |
| Examiner 2 measurement 2 | | -0.62 | <0.001 |
| Examiner 3 measurement 1 | | -0.38 | 0.032 |
| Examiner 3 measurement 2 | | -0.42 | 0.019 |
| WGS App weight shift to affected side versus 3DGA pelvic obl ROM affected side | | | |
| Examiner 1 measurement 1 | | -0.15 | 0.416 |
| Examiner 1 measurement 2 | | -0.11 | 0.543 |
| Examiner 2 measurement 1 | | -0.01 | 0.942 |
| Examiner 2 measurement 2 | | -0.06 | 0.760 |
| Examiner 3 measurement 1 | | -0.04 | 0.816 |
| Examiner 3 measurement 2 | | 0.02 | 0.923 |
| WGS App stance width versus 3DGA step width [m] | | | |
| Examiner 1 measurement 1 | | 0.34 | 0.032 |
| Examiner 1 measurement 2 | | 0.42 | 0.011 |
| Examiner 2 measurement 1 | | 0.42 | 0.011 |
| Examiner 2 measurement 2 | | 0.40 | 0.025 |
| Examiner 3 measurement 1 | | 0.40 | 0.025 |
| Examiner 3 measurement 2 | | 0.34 | 0.032 |
| WGS App guardedness versus 3DGA stance time [s] unaffected side | | | |
| Examiner 1 measurement 1 | | 0.12 | 0.497 |
| Examiner 1 measurement 2 | | 0.09 | 0.604 |
| Examiner 2 measurement 1 | | 0.06 | 0.736 |
| Examiner 2 measurement 2 | | 0.08 | 0.659 |
| Examiner 3 measurement 1 | | 0.06 | 0.727 |
| Examiner 3 measurement 2 | | 0.03 | 0.873 |
| WGS App a hip extension affected side versus 3DGA hip FE ROM affected side | | | |
| Examiner 1 measurement 1 | | -0.62 | <0.001 |
| Examiner 1 measurement 2 | | -0.38 | 0.032 |
| Examiner 2 measurement 1 | | -0.42 | 0.019 |
| Examiner 2 measurement 2 | | -0.55 | <0.001 |
| Examiner 3 measurement 1 | | -0.50 | <0.001 |
| Examiner 3 measurement 2 | | -0.42 | 0.019 |
| WGS App external rotation during initial swing affected side versus 3DGA hip IE ROM affected side | | | |
| Examiner 1 measurement 1 | | 0.74 | <0.001 |
| Examiner 1 measurement 2 | | 0.74 | <0.001 |
| Examiner 2 measurement 1 | | 0.65 | <0.001 |
| Examiner 2 measurement 2 | | 0.68 | <0.001 |
| Examiner 3 measurement 1 | | 0.65 | <0.001 |
| Examiner 3 measurement 2 | | 0.65 | <0.001 |
| WGS App circumduction at mid swing affected side versus 3DGA hip AA ROM affected side | | | |
| Examiner 1 measurement 1 | | 0.68 | <0.001 |
| Examiner 1 measurement 2 | | 0.74 | <0.001 |
| Examiner 2 measurement 1 | | 0.65 | <0.001 |
| Examiner 2 measurement 2 | | 0.65 | <0.001 |
| Examiner 3 measurement 1 | | 0.68 | <0.001 |
| Examiner 3 measurement 2 | | 0.42 | 0.011 |
| WGS App hip hiking at mid swing affected side versus 3DGA hip FE ROM affected side | | | |
| Examiner 1 measurement 1 | | -0.62 | <0.001 |
| Examiner 1 measurement 2 | | -0.62 | <0.001 |
| Examiner 2 measurement 1 | | -0.62 | <0.001 |
| Examiner 2 measurement 2 | | -0.62 | <0.001 |
| Examiner 3 measurement 1 | | -0.62 | <0.001 |
| Examiner 3 measurement 2 | | -0.55 | <0.001 |
| WGS App hip hiking at mid swing affected side versus 3DGA pelvic obl ROM affected side | | | |
| Examiner 1 measurement 1 | | -0.23 | 0.191 |
| Examiner 1 measurement 2 | | -0.23 | 0.191 |
| Examiner 2 measurement 1 | | -0.23 | 0.191 |
| Examiner 2 measurement 2 | | -0.23 | 0.191 |
| Examiner 3 measurement 1 | | -0.15 | 0.395 |
| Examiner 3 measurement 2 | | -0.16 | 0.383 |
| WGS App knee flexion from toe off to mid swing affected side versus 3DGA knee FE ROM affected side | | | |
| Examiner 1 measurement 1 | | -0.62 | <0.001 |
| Examiner 1 measurement 2 | | -0.62 | <0.001 |
| Examiner 2 measurement 1 | | -0.62 | <0.001 |
| Examiner 2 measurement 2 | | -0.62 | <0.001 |
| Examiner 3 measurement 1 | | -0.62 | <0.001 |
| Examiner 3 measurement 2 | | -0.62 | <0.001 |
| WGS App toe clearance affected side versus 3DGA total ankle flex IC and ankle flex TO affected side | | | |
| Examiner 1 measurement 1 | | -0.62 | <0.001 |
| Examiner 1 measurement 2 | | -0.62 | <0.001 |
| Examiner 2 measurement 1 | | -0.62 | <0.001 |
| Examiner 2 measurement 2 | | -0.62 | <0.001 |
| Examiner 3 measurement 1 | | -0.62 | <0.001 |
| Examiner 3 measurement 2 | | -0.62 | <0.001 |
| WGS App pelvic rotation at terminal swing affected side versus 3DGA pelvic rot ROM affected side | | | |
| Examiner 1 measurement 1 | | -0.31 | 0.025 |
| Examiner 1 measurement 2 | | -0.31 | 0.025 |
| Examiner 2 measurement 1 | | -0.31 | 0.025 |
| Examiner 2 measurement 2 | | -0.31 | 0.025 |
| Examiner 3 measurement 1 | | -0.31 | 0.025 |
| Examiner 3 measurement 2 | | -0.31 | 0.025 |
| WGS App initial foot contact affected side versus 3DGA ankle flex IC affected side | | | |
| Examiner 1 measurement 1 | | -0.62 | <0.001 |
| Examiner 1 measurement 2 | | -0.62 | <0.001 |
| Examiner 2 measurement 1 | | -0.62 | <0.001 |
| Examiner 2 measurement 2 | | -0.62 | <0.001 |
| Examiner 3 measurement 1 | | -0.62 | <0.001 |
| Examiner 3 measurement 2 | | -0.62 | <0.001 |

R – value of Spearman rank correlation test; R^2^ – value of Spearman’s coefficient of determination, p – probability values, Pelvic obl ROM - pelvic oblique range of motion, Hip FE ROM - hip flexion/extension range of motion, Hip IE ROM - hip internal/external rotation range of motion, Hip AA ROM - hip abduction/adduction range of motion, Knee FE ROM – knee flexion/extension range of motion, Ankle flex IC - ankle flex in initial contact phase, Ankle flex TO - ankle flex in toe off phase, Pelvic rot ROM - pelvic rotation range of motion
